# Trends in Internet Searches for Cannabidiol (CBD) in the United States

**DOI:** 10.1001/jamanetworkopen.2019.13853

**Published:** 2019-10-23

**Authors:** Eric C. Leas, Alicia L. Nobles, Theodore L. Caputi, Mark Dredze, Davey M. Smith, John W. Ayers

**Affiliations:** 1 Division of Health Policy, Department of Family Medicine and Public Health, University of California, San Diego, La Jolla; 2 Division of Infectious Diseases and Global Public Health, Department of Medicine, University of California, San Diego, La Jolla; 3 Department of Health Sciences, University of York, York, United Kingdom; 4 Department of Computer Science, Johns Hopkins University, Baltimore, Maryland

## Abstract

This cross-sectional study examines Google searches for cannabidiol (CBD) in the United States to gauge public interest in the use of CBD.

## Introduction

Cannabidiol (CBD) is widely promoted as a panacea. For example, the cannabis brand MedMen claims CBD treats acne, anxiety, opioid addiction, pain, and menstrual problems.^1^ However, the US Food and Drug Administration has only approved highly purified CBD (Epidiolex) for treating epilepsy. To our knowledge, there is currently no population-focused surveillance of public interest in CBD. Consequently, many question whether CBD should be prioritized by public health leaders and regulators. This article describes public interest in CBD within the United States.

## Methods

In this longitudinal cross-sectional study, we measured Google searches that mentioned “CBD” or “cannabidiol” emerging from the United States (including by state) from January 1, 2004, through April 23, 2019. We report these measurements as query fractions (QFs), which are the number of searches per every 10 million searches originating nationally or from a respective state. We forecasted searches through 2019 using an autoregressive integrated moving average model, with components estimated using the algorithm developed by Hyndman and Khandakar^2^ and data from all months since 2014. This model is robust to time series biases such as recurring periodicities. We also contrasted search volumes during April 2019 for CBD against those for several health topics, products, or alternative medicines, including acupuncture, apple cider vinegar, diet (excluding “Coke” or “Pepsi”), electronic cigarettes (“e cig/s,” “e cigarette/s,” “electronic cigarette/s,” “vape,” “vaping,” or “vapor,” excluding “marijuana” or “cannabis”), exercise, marijuana (“marijuana” or “cannabis”), meditation, vaccines (“vaccine/s,” “vaccinate/d,” or “vaccination”), veganism, and yoga. Raw search counts were inferred using estimates from the media measurement and analytics company Comscore. Analyses were performed using R statistical software version 3.5.2 (R Project for Statistical Computing) and followed the Strengthening the Reporting of Observational Studies in Epidemiology (STROBE) reporting guideline for cross-sectional studies. Ethical review by the institutional review board was not required per 45 CFR part 46 and the standard operating policies and procedures at the University of California, San Diego, which do not require review of studies that do not involve intervention or interaction with individuals and use information that is not individually identifiable.

## Results

**Figure 1.**
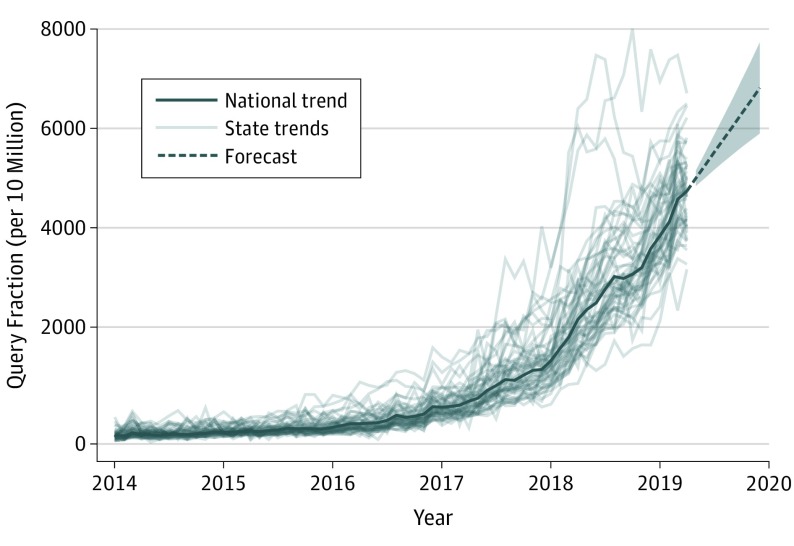
National and State Google Searches for Cannabidiol or CBD Since 2014 Forecasts were projected through 2019 using the forecasting algorithm developed by Hyndman and Khandakar.^2^

Nationally, CBD searches were stable from 2004 through 2014 but then substantially increased (Figure 1). Search volumes increased 125.9% during 2017 compared with 2016, 160.4% during 2018 compared with 2017, and are expected to be 117.7% (95% prediction interval, 99.5%-135.6%) higher during 2019 than 2018 based on observed and forecasted volumes. There were 6.4 million CBD Google searches during April 2019, the last month data were collected.

The increase in CBD searches encompassed all states, ranging from a 211.2% increase (2014-2018 to 2019) in Oklahoma to a 605.0% increase in Alabama. During 2019 (January to April), Vermont (QF = 7164.1), Wisconsin (QF = 6120.6), Tennessee (QF = 6096.2), Colorado (QF = 5815.5), New Hampshire (QF = 5391.4), and Oregon (QF = 5369.2) had the most searches. Searches were higher during 2019 in states that had legalized recreational marijuana (mean [SD] QF = 4975.6 [814.0]) than in states with medicinal marijuana (mean [SD] QF = 4451.9 [609.7]) or marijuana prohibitions (mean [SD] QF = 4560.9 [964.0]).

**Figure 2.**
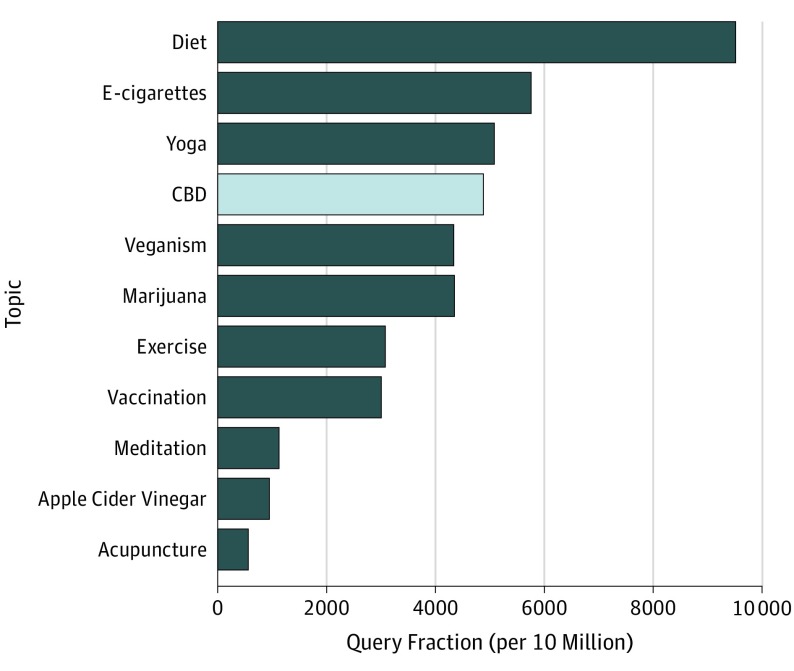
Google Searches for Cannabidiol or CBD and for Other Health Topics in April 2019 Comparison of April 2019 Google searches that included the terms *cannabidiol* or *CBD *with searches including terms for other health topics*. *Specific lists of queries are given in the Methods section. e-Cigarettes indicates electronic cigarettes.

Searches for CBD during April 2019 eclipsed those for acupuncture by a factor of 7.49, apple cider vinegar by 5.17, meditation by 3.38, vaccination by 1.63, exercise by 1.59, marijuana by 1.13, and veganism by 1.12 (Figure 2). Searches for CBD are now rivaling searches for yoga and electronic cigarettes, with 0.96 and 0.85 of their respective search volumes, and are searched for more than half as much as dieting (0.51).

## Discussion

The findings of this longitudinal cross-sectional study indicate that interest in CBD across the United States has increased considerably and is accelerating. While our study is limited in that Google searches may reflect interest in CBD rather than interest in use, search trends are associated with many health-related behaviors,^3^ including the rise of electronic cigarettes,^4^ years ahead of traditional data. Thus, our findings suggest that investigation into CBD should become a public health priority to catch up with the public’s interest.

First, studies should focus on the epidemiology of CBD use, characterizing who uses CBD products and for what purposes. Second, the effects and potential drug interactions of CBD should be evaluated. Third, given that CBD products are often mislabeled^5^ and adulterated products have led to mass poisonings,^6^ product safety standards must be developed. Fourth, marketing practices around CBD should be standardized, as marketing that misleads the public could erode trust in evidence-based medicine.
